# Ion Channel Gene Mutations Causing Skeletal Muscle Disorders: Pathomechanisms and Opportunities for Therapy

**DOI:** 10.3390/cells10061521

**Published:** 2021-06-16

**Authors:** Lorenzo Maggi, Silvia Bonanno, Concetta Altamura, Jean-François Desaphy

**Affiliations:** 1 Neuroimmunology and Neuromuscular Disorders Unit, Fondazione IRCCS Istituto Neurologico Carlo Besta, 20133 Milan, Italy; silvia.bonanno@istituto-besta.it; 2 Department of Biomedical Sciences and Human Oncology, School of Medicine, University of Bari Aldo Moro, 70124 Bari, Italy; concetta.altamura@uniba.it (C.A.); jeanfrancois.desaphy@uniba.it (J.-F.D.)

**Keywords:** ion channels, myotonia, periodic paralysis, myopathies, SCN4A, CACNA1S, CLCN1, KCNJ2

## Abstract

Skeletal muscle ion channelopathies (SMICs) are a large heterogeneous group of rare genetic disorders caused by mutations in genes encoding ion channel subunits in the skeletal muscle mainly characterized by myotonia or periodic paralysis, potentially resulting in long-term disabilities. However, with the development of new molecular technologies, new genes and new phenotypes, including progressive myopathies, have been recently discovered, markedly increasing the complexity in the field. In this regard, new advances in SMICs show a less conventional role of ion channels in muscle cell division, proliferation, differentiation, and survival. Hence, SMICs represent an expanding and exciting field. Here, we review current knowledge of SMICs, with a description of their clinical phenotypes, cellular and molecular pathomechanisms, and available treatments.

## 1. Introduction

Primary ion channelopathies are rare diseases caused by mutations in genes encoding ion channel subunits. The first ion channelopathy was identified in hyperkalemic periodic paralysis (hyperPP), a genetic disease affecting exclusively skeletal muscles and caused by mutations in the *SCN4A* gene encoding the alpha subunit of voltage-gated sodium channel Nav1.4, which expression is limited to the skeletal muscle fiberfibers [1]. The discovery of *CFTR* gene mutations linked to cystic fibrosis was contemporaneous, but the chloride channel activity of the CFTR protein was not known yet. Thus, skeletal muscle ion channelopathies (SMICs) have been a paradigm for the discovery of ion channelopathies affecting all organs. Considering the importance of ion channels in modulating membrane electrical activity, the diseases related to ion channel mutations were characterized by disturbance of muscle fiber excitability, such as the non-dystrophic myotonias (NDM) presenting with muscle stiffness (myotonia) due to membrane over-excitability and the periodic paralysis (PP) showing episodes of paralysis due to sarcolemma inexcitability.

Yet, with the development of more advanced technologies, including next-generation and whole-exome sequencing, it appears more and more evident that ion channel mutations may cause further muscle phenotypes, including progressive myopathies, altering muscle structure, thereby highlighting a significant role of ion channels in muscle cell division, proliferation, differentiation, and survival.

Hence, SMICs represent a large heterogeneous group of rare genetic disorders resulting in long-term disabilities with a relevant burden to the patients, families and National Health Care Services. SMICs usually present in childhood, but late-onset cases have been reported. SMICs diagnosis requires a high clinical suspicion, being mainly based on the detailed clinical history and neurological examination, followed by molecular confirmation.

Here, we review current knowledge of SMICs, with a description of clinical phenotype, cellular and molecular pathomechanisms, and available therapies. We also included the description of two neuronal ion channelopathies (*TRPV4* and *KCNA1* genes), which may have pronounced effects on skeletal muscles. Ion channel gene mutations and related clinical muscular phenotypes are summarized in Table 1.

## 2. Skeletal Muscle Sodium Channelopathies

**Figure 1:**
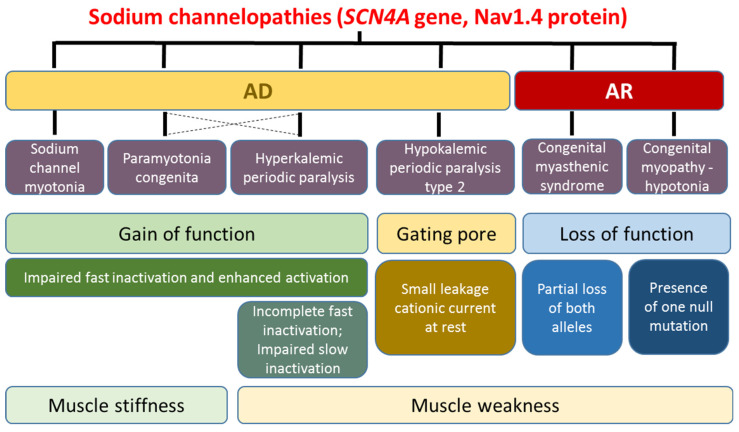
A schematic representation of skeletal muscle sodium channelopathies, reporting the mode of inheritance (AD: autosomal dominant; AR: autosomal recessive), disease name, mutation effects on sodium channel function, and main symptoms.

Voltage-gated sodium channels are responsible for the upstroke of action potentials and modulate firing frequency in excitable cells. From the first week of life, the main sodium channel expressed in skeletal muscles is Nav1.4, which alpha-subunit is encoded by *SCN4A*. The spectrum of SCN4A-related myopathy spans from the complete loss of the channel (null mutations) to a gain of function (missense mutations) through various degrees of altered function, resulting in highly heterogeneous clinical presentation, mainly characterized by myotonia or PP, which represents a continuum in the clinical spectrum (Figure 1). In particular, the most frequent phenotype is paramyotonia congenita (PMC), followed by sodium channel myotonia (SCM), while PP is less frequent [2,3,4].

### 2.1. Hyperkalemic Periodic Paralysis, Paramyotonia Congenita, and Sodium Channel Myotonia

The *SCN4A* mutations were first identified in patients suffering from the autosomal dominant hyperPP [1,5,6]. HyperPP is characterized by episodes of flaccid paralysis, leading to muscle weakness, generally associated with ictal hyperkalemia (>4.5 mEq/L). The paralytic attack can last for up to 2 h. Loading of K^+^ can provoke or worsen an attack; other triggers include rest after exercise, fasting, and cold exposure. Emotional stress and pregnancy can increase the likelihood of attacks. The first attack is generally experienced during the first decade of life. Permanent weakness may progressively take place after the fourth decade. Myotonia is experienced by many hyperPP patients [4,7].

PMC is allelic to hyperPP, being caused by autosomal dominant *SCN4A* missense mutations [8]. PMC is mainly characterized by paradoxical myotonia that typically worsens with exercise and shows no warm-up phenomenon, which is usually detected in myotonia congenita (MC). In addition, episodes of flaccid paralysis can represent a relevant feature in patients with PMC [4,9]. Triggers of myotonia are similar to those reported in hyperPP. Thus, PMC and hyperPP are considered as a continuum manifesting as pure PMC, pure hyperPP, or intermediary PMC plus hyperPP. Other *SCN4A* mutations are responsible for SCM, which can be distinguished from PMC by the lack of both paradoxical myotonia and episodes of flaccid paralysis [10]. In addition, PMC differs from SCM for earlier onset, higher cold-sensitivity, and more frequent involvement of hand and cranial muscles [4,9,11]. Symptoms in SCM are quite variable in severity and may show specific features; thus, various subgroups have been described, such as myotonia fluctuans (moderate), myotonia permanens (severe), acetazolamide-responsive myotonia, potassium-aggravated myotonia, and painful myotonia. In addition, myotonia permanens can be associated with harmful neonatal symptoms, such as severe neonatal episodic laryngospasm (SNEL) [12].

It needs to be noted that some mutations can lead to different phenotypes, even in the same kindred. Again, this argues for a continuum of these disorders and suggests the importance of disease-modifying genes. As more mutations are identified, many variable phenotypes are emerging, such as myotonia with normokalemic/hypokalemic PP, PP associated with myotonia instead of paradoxical myotonia, or the presence of myopathic traits [13,14,15,16,17,18].

Up to date, about seventy missense *SCN4A* mutations were found to be linked to these diseases, half of which were functionally characterized. Patch-clamp and computational experiments have clearly demonstrated that myotonia is due to a gain of function of the mutated Nav1.4 channel, mainly through the defects of inactivation and enhancement of activation [19]. Thus, the mutated channel activates more quickly or inactivates more slowly or incompletely, as compared to the wild-type channel. Recovery from inactivation can be accelerated. The voltage dependence of activation and inactivation can also be shifted toward potentials favoring channel activity [19]. These effects may be temperature-dependent, in accord with the cold sensitivity of the patients. The resulting increased influx of sodium ions rends the muscle fibers more excitable and inclined to generate high-frequency firing of action potentials that persists after the end of motoneuron stimulation. Such after-discharges cause a delay in relaxation and muscle stiffness. Spontaneous firing can also occur in the absence of a nervous stimulus, which can induce muscle spasms, fasciculation, and cramps. Continuous activity can induce muscle hypertrophy.

The voltage-gated sodium channels can enter two inactivated states: the fast one normally occurring on a millisecond time scale and the slow one occurring on a second time scale. The mutations that produce a persistent inward sodium current due to incomplete fast inactivation and impair Nav1.4 slow inactivation are more inclined to induce PP than myotonia [20,21]. In these conditions, the persistent depolarization of sarcolemma may, in turn, inactivate the wild-type sodium channels, rendering the cell inexcitable with flaccid paralysis.

Treatment of myotonia relies on the use of frequency-dependent sodium channel blockers that reduce excessive action potential firing in the overexcited muscle fibers. The cardiac antiarrhythmic, mexiletine, has been granted as an orphan drug for myotonic syndromes after the success of randomized clinical trials (RCT) and is widely recognized as the first drug of choice in myotonia [22,23]. However, up to 30% of myotonic patients have an unsatisfactory response to mexiletine because of a suboptimal response or side effects [24,25]. The antiepileptic drug lamotrigine was beneficial in an RCT, and it is now considered the second choice in several countries [26,27]. Moreover, the antiarrhythmic flecainide and propafenone were proved useful in mexiletine-refractory patients carrying specific *SCN4A* mutations, suggesting the possibility of defining a mutation-driven pharmacological strategy [28,29,30,31]. It is feasible to introduce the marketed sodium channel blockers for repurposing myotonia [32,33,34,35] or developing new derivatives with increased efficacy [36,37].

Intuitively, the use of sodium channel blockers would not be recommended in hyperPP because they might worsen the fiber inexcitability. Intravenous insulin/glucose or inhaled salbutamol can be used to abort a paralytic attack because they induce the hyperpolarization of muscle membrane through stimulation of Na^+^-K^+^ pump. Prophylaxis of recurrent attacks is based on the use of carbonic anhydrase inhibitors (CAI), acetazolamide (ACZ) or dichlorphenamide, and/or thiazide diuretics to maintain the low blood level of potassium. The CAI can enhance the activity of Ca^2+^-activated K^+^ (BK) channels and ClC-1 chloride channels in muscle membranes [38,39]. The benefits of dichlorphenamide in hyperPP were confirmed in an RCT [40]. Recommendations and guidelines on diagnosis and treatment of NDM and PP were previously detailed by Stunnenberg et al. and Statland et al., respectively [41,42].

### 2.2. Hypokalemic Periodic Paralysis Type 2

Hypokalemic PP type 2 (hypoPP2) is a distinct syndrome caused by the missense *SCN4A* mutations [43,44]. It is characterized by episodes of focal (limb) or more frequently generalized flaccid paralysis, with a longer duration than hyperPP, lasting more than 2 h up to days, with concomitant hypokalemia (<3.5 mEq/L), and absence of myotonia [4,7,41]. The attack triggers include carbohydrate or alcohol ingestion and rest after vigorous exercise. The disease onset is variable, occurring in between the first and third decade of life. The frequency and severity of attacks generally decrease after the third decade, but permanent weakness and progressive myopathy may develop in the proximal muscles of the lower limbs.

Ten *SCN4A* mutations have been linked to hypoPP2, which accounts for about 20% of all hypoPP cases. Indeed, around 60% of hypoPP cases are caused by *CACNA1S* mutations (hypoPP type 1, hypoPP1) [2,3]. All the hypoPP are inherited with an autosomal dominant trait. Interestingly, most *SCN4A* and *CACNA1S* hypoPP mutations share a similar molecular defect [45]. These mutations are all sited in the voltage-sensing S4 segments of the channel, neutralizing a positive charge through the substitution of an arginine residue [45]. Functional studies suggested that the mutations induce various degrees of loss of function, but it appeared that the most likely pathomechanism consists in the creation of an aberrant pore beside the normal ion conduction pathway [45]. This so-called gating pore permits small leakage of cationic currents at rest, favoring membrane depolarization in hypokalemia that, in turn, inactivates sodium channels and rends the fiber inexcitable.

Oral or intravenous potassium loading is the only way to abort a paralytic attack in hypoPP. For prophylaxis, the use of ACZ or dichlorphenamide is the preferred strategy. Their mechanism of action in hypoPP is not well defined but might involve systemic acidosis and BK channel activation. The K^+^-sparing diuretics, such as eplerenone or spironolactone, can be used in addition or alternative to CAI. Evidence has been provided that suggests a relationship between genotype and response to CAI, with hypoPP2 often less responsive than hypoPP1 and arginine-to-histidine mutations more responsive than other mutations, highlighting again the importance of genotyping for better addressing therapy [46].

### 2.3. Congenital Myasthenia and Congenital Myopathy Related to SCN4A

Very rare *SCN4A* mutations producing a loss of function of Nav1.4 channels have been associated with congenital disorders. They include congenital myasthenic syndrome (CMS) with brief and abrupt attacks of muscle weakness since birth, which may eventually lead to respiratory arrest; other mutations induce neonatal hypotonia and congenital myopathy; finally, some mutations may produce severe fetal hypokinesia leading to death in utero or soon after birth [47,48]. These mutations are inherited in an autosomal recessive mode. The presence of two mutations producing a partial loss of Nav1.4 function leads to congenital myasthenia. A heterozygous null mutation in the *SCN4A* gene is associated with neonatal hypotonia and congenital myopathy; the concomitance of two null mutations induces neonatal death [19,33]. ACZ has provided some benefits in some patients, but its mechanism of action is still unknown [49,50].

## 3. Calcium Channel-Related Myopathies

**Figure 2:**
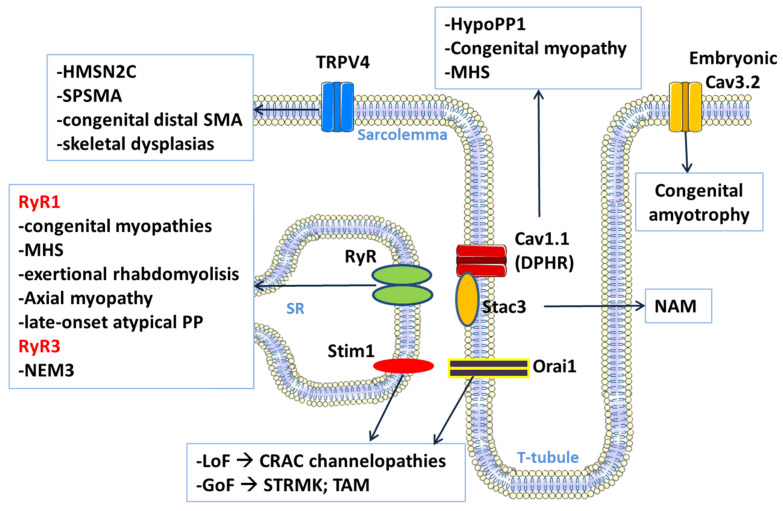
A schematic representation of the calcium ion-permeable channels involved in skeletal muscle myopathies. The TRPV4 channel is highly expressed even in motor neurons, and mutations cause a number of peripheral neuropathies with implications for skeletal muscles. The contribution of muscular TRPV4 channels to these diseases is unknown. The Cav3.2 channel is expressed mainly in embryonic cells, which contributes to myoblast differentiation and fusion during muscle development. (CRAC: Calcium-release activated calcium; DHPR: Dihydropyridine receptor; GoF: gain of function; HMSN2C: Hereditary motor and sensory neuropathy type 2C; HypoPP1: Hypokalemic periodic paralysis type 1; LoF: loss of function; MHS: Malignant hyperthermia susceptibility; NAM: Native American myopathy; NEM3: Nemaline myopathy type 3; SMA: Spinal muscular atrophy; SPSMA: Scapuloperoneal spinal muscular atrophy; SR: sarcoplasmic reticulum; STRMK: Stormorken syndrome; TAM: Tubular aggregate myopathy).

Calcium ions play a central role in regulating muscle function. In the short term, calcium ions released by the sarcoplasmic reticulum (SR) in response to electrical excitation of the cell membrane trigger muscle fiber contraction. In the long term, calcium ions modulate gene expression, contributing to muscle plasticity, which is the ability of muscle fibers to adapt their mass and metabolism to functional needs. Thus, calcium ion-permeable channels and associated proteins are critical for the maintenance of the contraction apparatus of muscle. Deleterious mutations in these proteins cause diseases characterized by alteration of excitability, excitation–contraction coupling, and skeletal muscle structure (Figure 2).

### 3.1. CACNA1S-Related Disorders

The *CACNA1S* gene codes for the CaV1.1 channel, the pore-forming subunit of the dihydropyridine receptor (DHPR), a voltage-gated L-type Ca^2+^ channel located on the T-tubule, which activates the type 1 ryanodine receptor (Ryr1) calcium channel sited in the SR, during excitation–contraction coupling.

Different phenotypes were reported in patients with mutations in *CACNA1S*, such as hypoPP1, congenital myopathy, and malignant hyperthermia susceptibility (MHS).

#### 3.1.1. Hypokalemic Periodic Paralysis Type 1

The main phenotype caused by autosomal dominant mutations in *CACNA1S* is hypoPP1) [51]. The frequency of attacks is highly variable, from daily to once in a lifetime. The age of onset is usually earlier, and the duration of paralytic episodes longer in hypoPP1 than hypoPP2 [7]. Muscle strength is normal between attacks; however, permanent muscle weakness, regardless of the episodes of paralysis, is frequently observed in later disease stages of hypoPP1, while it is not reported in hypoPP2. In this regard, a recent paper on a large cohort of hypoPP1 patients showed that at least one-third developed permanent weakness with ageing [52].

Carbohydrate load and rest after a strenuous exercise are the most common triggers for hypoPP, although attacks may occur spontaneously. Muscle biopsy in hypoPP1 may show non-rimmed vacuoles, whereas, in hypoPP2, vacuoles occur as frequently as tubular aggregates [7]. However, hypoPP diagnosis is essentially based on medical history and then confirmed by molecular analysis. ACZ is the most common treatment choice for hypoPP, in particular for type 1 [46]. Recently, two randomized, double-blind, placebo-controlled trials showed a reduction of attack frequency with dichlorphenamide [40].

Missense mutations causing hypoPP affect highly conserved arginine residues of the S4 voltage-sensing domains of the CaV1.1, resulting in aberrant gating pore currents leading to paradoxical depolarization of the membrane in low potassium conditions and inexcitability [45,53,54].

Two mutations, p.R528H and p.R1239H, occur in nearly 80% of patients with hypoPP1 in England and The Netherlands [2,3].

#### 3.1.2. Calcium Channel-Related Congenital Myopathy

Autosomal dominant or recessive mutations in *CACNA1S* have been detected through exome sequencing in seven families manifesting a congenital myopathy [55]. Patients presented with antenatal/congenital or early-onset generalized, predominantly axial muscle weakness. Ophthalmoplegia was detected in about one-third of the patients. Notably, one patient also developed PP. Histological analysis showed features resembling core and centronuclear myopathies. Taken together, all these features were suggestive of congenital myopathies. Notably, congenital myopathies were mostly considered as disorders of excitation–contraction coupling and impaired calcium handling [56]. There is no genotype–phenotype correlation in those patients with CaV1.1 mutation-induced congenital myopathy. Both dominant and recessive mutations caused a decrease in DHPR function in skeletal muscle, with a decrease in protein level and a relevant impairment of Ca^2+^ release induced by the sarcolemma depolarization in cultured myotubes. A further case of congenital myopathy with ophthalmoplegia has been reported by Hunter and collaborators [57]; two additional severe cases with fetal akinesia leading to premature termination of pregnancy at 26 weeks of gestation or death after 10 days of were reported by Ravenscroft and collaborators [58]. Three Turkish siblings carrying homozygous *CACNA1S* mutation presented a congenital myopathy with additional features, including cognitive delay, pes equinovarus deformity, and neurogenic changes at muscle biopsy [59].

### 3.2. STAC3-Related Disorder

EC coupling in skeletal muscles operates through the triad junctions, where the voltage sensor Ca_V_1.1, through its β_1a_ subunit, physically interacts with the Ryr1 calcium release channel. Recently, the STAC3 (SH3 and cysteine-rich domain 3) protein consisting of three scaffold proteins was found to be another key component of the skeletal muscle EC coupling [60]. *STAC3* is specifically expressed in skeletal muscles, where it binds to Cav1.1 in the triads, and its inactivation generates skeletal muscle paralysis within the framework of conserved muscle differentiation and NMJ formation, indicating that the mechanism is altered downstream muscle excitation [61,62]. Recently, a homozygous missense mutation, p.W284S of human Stac3 (c.1046G>C), was identified in a cohort of five families (5 individuals affected and 13 unaffected) presenting a rare autosomal recessive congenital myopathy [62]. The disease was initially described in 21 Lumbee Native Americans, thus called “NAM,” Native American Myopathy, also known as Bailey-Bloch congenital myopathy (MYPBB) [63,64,65]. The symptoms of NAM usually occur at birth or in the first years of life with facial involvement (ptosis, downturned corners of the mouth, cleft palate), bone and joint deformities (arthrogryposis, scoliosis, and short stature), MHS, congenital weakness, and delayed motor milestones. Premature death occurs by the age of 18 in 6%–30% of the cases [62,66]. Recently, the homozygous p.W284S mutation was found in 2 siblings from Qatar, and the compound heterozygous for the p.W284S and a 4-bp deletion was described in other two siblings from Puerto Rico [67], all of the patients exhibiting the NAM phenotype. In addition, 18 patients of African, Middle Eastern, and South American ancestry presenting symptoms ranging from severe prenatal/neonatal onset to slowly progressive congenital myopathy (6 months–22 years old) were found homozygous or compound heterozygous for the p.W84S mutation. Thus, the p.W284S mutation is not restricted to the Native American population [68]. The first NAM-affected patient of Turkish origin carries two heterozygous variants (c.862A>T; p.K288* and c.432+4A>T), close to but not into the p.W284S region of the STAC3 gene [69]. The STAC3 SH3-1 domain is highly conserved throughout species and is the site of all the NAM causative Stac3 variants to date [66,70]. This domain interacts with the II-III loop Cav1.1 and is crucial for EC coupling. Several in vitro and in vivo studies demonstrated that Stac3 operates as a chaperone for CaV1.1 and modulates the L-type calcium currents [60]. Lastly, it has been speculated that Stac3 directly binds to Ryr1, which possibly constitutes the long-sought physical link between CaV1.1 and Ryr1 [71]. Yet, further evidence is needed to demonstrate the existence of this binding site and its therapeutic implications in CaV1.1/ Ryr1/STAC3 channelopathies.

### 3.3. STIM1 and ORAI1-Related Disorders

Store-operated Ca^2+^ entry (SOCE) is the process by which calcium depletion in the endoplasmic or sarcoplasmic reticulum stimulates extracellular Ca^2+^ influx through the plasma membrane [72]. This calcium current is mediated by the calcium release-activated calcium modulator 1 (ORAI1) and the stromal interaction molecule type 1 (STIM1), which constitute the Ca^2+^ release-activated Ca^2+^ (CRAC) channels [73,74]. ORAI1 is a four alpha-helical transmembrane protein forming the pore unit in the sarcolemma; its activation is mediated by the direct interaction with STIM1, a single-span transmembrane protein situated on the SR membrane where it senses Ca^2+^ depletion [75,76]. Regulation and function of the CRAC channel have been largely investigated, highlighting their relevance in fundamental cellular processes such as gene transcription, cellular metabolism, vesicle trafficking, muscle contraction, and myoblasts development [77,78,79]. Due to their crucial role in calcium signaling, mutations in *STIM1* and *ORAI1* are associated with abnormal SOCE and multi-systemic clinical phenotypes, ranging from immune system dysregulation to hematological defects and, notably, different forms of muscle involvement depending on the loss- or gain-of-function mutations of the genes.

Loss-of-function mutations in *ORAI1* and *STIM1* result in suppressed protein expression, thus abolishing SOCE. They are very rare, all inherited in an autosomal recessive manner, and constitute the “CRAC channelopathies” that present with a homogeneous syndromic phenotype characterized by neonatal immunodeficiency with recurrent severe infections due to defective lymphocyte activation, ectodermal dysplasia (altered tooth enamel, anhidrosis), autoimmune neutropenia and thrombocytopenia, encephalopathy and seizures, along with congenital non-progressive muscular hypotonia. The myopathy in patients with *ORAI 1* and *STIM 1* loss-of-function mutations is characterized by delayed motor milestones, proximal lower limb weakness, nasal speech, generalized hypotonia with atrophy of type 2 fibers, and reduced fatigue resistance [74]. Missense mutations in *ORAI1* impair but not abolish ORAI1 protein expression [80,81]; however, they cause a severe clinical CRAC channelopathy spectrum. In contrast, missense mutations in the C-terminus of *STIM1*, which may allow a minimal residual SOCE, present with a later onset and a milder phenotype [82,83].

Contrarily to loss-of-function mutations, different gain-of-function (GoF) mutations in *ORAI1* and *STIM1* result in the activation and increased SOCE. They are inherited in an autosomal dominant manner, constituting rare clinical phenotypes with a wide disease spectrum. The complex Stormorken syndrome (STRMK) is characterized by bleeding diathesis, thrombocytopenia, congenital miosis, ichthyosis, asplenia, tubular aggregate myopathy (TAM), small stature with syndromic facial features, and cognitive impairment [84,85]. However, GoF mutation in ORAI1 and STIM1 might also result in the York platelet syndrome [86], showing thrombocytopenia, bleeding diathesis, and muscle weakness until isolated TAM [87]. TAM is a progressive muscle disorder characterized by subsarcolemmal basophilic inclusions, stained red on modified Gomori trichrome, positive to NADH and negative for SDH reactions [88]. It remains unclear how tubular aggregates (TA) form, but it has been suggested that constitutive Ca^2+^ loading may result in misfolding and aggregation of SR proteins [89], especially in type II atrophic fibers [90]. Tubular aggregates are not specific for TAM, as they may also appear in congenital myasthenic syndrome caused by *DPAGT1* and *GFPT1* mutations [91,92], in hypoPP [93]. *STIM1* is the major gene responsible for TAM (TAM type 1). STIM1-EF hands at the N-terminus coordinate Ca^2+^-binding and maintain STIM1 in an inactive conformation. At least eight mutations in the EF hands have been reported in the His109 and Phe108 residues, a potential TAM mutation hotspot [94,95,96,97]. TAM phenotype is clinical heterogeneous ranging from slowly progressive proximal muscle weakness predominantly affecting the lower limbs, with adult onset (>35 year), to myalgia only. Serum creatine kinase is usually elevated, and patients may develop eye movement disabilities and joint contractures during disease progression [74,94,98]. Notably, the same *STIM1* mutations can also be responsible both for the STRMK [89,90,99] and the York syndrome [86], emphasizing how all these conditions represent a continuum. Slowly progressive TAM with elevated creatine phosphokinase (CPK) levels might be related even to GoF mutations in *ORAI1* (TAM type 2) transmembrane domains, which are sometimes accompanied by miosis constituting an STRMK-like syndrome [89,100,101,102].

A few CRAC channel modulators are currently in a clinical trial, mainly for psoriasis treatment [103]; the continuous advancement in the comprehension of the STIM1–ORAI1 interaction and molecular regulation is expected to underscore new potential therapeutic targets to modulate SOCE and restore aberrant Ca^2+^ homeostasis.

### 3.4. RYR1-Related Disorders

The *RYR1* gene is located on chromosome 19q13.1 and encodes RyR1, the principal sarcoplasmic reticulum calcium release channel playing a fundamental role in the EC coupling process [104].

RYR1-related myopathies represent the most common form of congenital myopathy [105]. Phenotypes associated with *Ryr1* gene mutations include a wide range of diseases with some overlaps, hence representing a continuum in the clinical spectrum [106,107,108,109,110,111,112]. Hence, a relevant issue in *Ryr1* gene analysis is the definition of pathogenicity of novel variants, in particular considering the great clinical variability associated with *RYR1* mutations. In this regard, the correlation of genetic findings together with clinical, histological, and muscle imaging data may be helpful. All these diseases show impaired intracellular calcium homeostasis through different pathomechanisms, such as leaky Ryr1 channels, reduced Ryr1 expression, impaired Ryr1 interdomain interactions, increased sensitivity to modulators/activators, or impaired excitation–contraction coupling [113]. The impaired calcium homeostasis may cause secondary cellular dysfunction with increased oxidative stress, abnormal post-translational modifications, mitochondrial dysfunction, and altered protein–protein and protein–ligand interactions [113].

#### 3.4.1. RYR1-Related Congenital Myopathies

Autosomal dominant mutations in *RYR1* have been associated with the core myopathies (CM), the most frequent congenital myopathy subtype. Most of the mutations are missense, with a few small deletions and duplications [114,115,116,117,118,119,120]. Core myopathies include central core disease (CCD) and multiminicore myopathy, characterized by the presentation of cores and minicores, respectively, in the muscle fibers and defined as distinct areas of myofibrillar disruption lacking mitochondria. Patients with dominant core myopathy typically present at birth or first years of life with muscle hypotonia, proximal weakness, mild cranial involvement, and orthopedic complications, as contractures, congenital hip dislocation and scoliosis [121,122].

More recently, recessive mutations in *RYR1* have been described in patients with CCD or centronuclear myopathy, another congenital myopathy subtype [106,121,123,124].

Mutations in *RYR1* were associated with the specific subtypes of congenital myopathy, such as congenital fiber-type disproportion and rod-core myopathy [123,125]. The frequency of recessive mutations was similar to that of dominant ones [106]. Dominant mutations are usually associated with milder phenotypes, whereas patients with recessive inheritance show earlier onset, more pronounced weakness and motor function disabilities. Bulbar and extraocular muscle involvement was almost exclusively reported in the recessive cases [106].

Although there are three hotspot regions (*N*-terminal residues 1–614, central residues 2163–2458, and *C*-terminal pore/transmembrane residues 4136–4973), autosomal dominant *RYR1* mutations in CCD and MHS may cover the entire gene [106,117,126]. In recessive *RYR1*-related myopathies, mutations are widespread across the entire *RYR*1 gene, usually identified as a combination of a null mutation and a missense mutation or two missense mutations, with the variable clinical severity [106,123,127,128].

Recently, an additional congenital myopathy phenotype has been described, which is characterized by irregular areas of myofibrillar disorganization with a reddish-purple granular material deposition with uneven oxidative stain and devoid of ATPase activity; this novel histological entity has been named dusty core myopathy (DCM) and is clinically indistinguishable from the aforementioned recessive core myopathy cases [111]. The authors of [111] suggested that DCM represents the most common CM associated with recessive *RYR1* mutations.

Fetal akinesia syndrome represents the most severe phenotype associated with RYR1 mutations; it has been reported in patients carrying autosomal dominant or recessive *RYR1* mutations and with histological diagnosis of CCD [129].

#### 3.4.2. Malignant Hyperthermia Susceptibility

MHS is characterized by muscle hypermetabolism that is triggered by anesthetic agents, particularly volatile gases (e.g., sevoflurane, desflurane) and depolarizing muscle relaxants (succinylcholine), which cause muscle rigidity and hyperthermia [130]. The incidence of MHS is estimated to be as high as one in 2000 Western Europeans, one event in every 50,000 anesthesia uses in adults, and one event in every 10,000 anesthesia uses in children. MHS represents a medical emergency and is lethal in about 70% of cases if not treated in time with supportive care measures and dantrolene, which antagonizes the excessive intracellular release of calcium by the Ryr1. Autosomal dominant mutations in *RYR1* represent by far the most frequent cause of MHS, followed by mutations in *CACNA1S*, accounting for about one-fourth of cases [131]; in addition, a few cases have been reported in association with mutations in the *STAC3* gene [132].

The in vitro contracture test on muscle biopsy tissue is the definitive diagnostic test for MHS. It cannot be routinely performed because it requires specific skills and is not possible in an emergency. In addition, false-positive diagnoses have been reported, and an underlying myopathy may produce a positive contraction test [133]. Genetic testing is also challenging, as about half of the MHS patients have no detected mutations in *RYR1*, *CACN1AS*, or *STAC3*.

The King Denborough syndrome, which is characterized by MHS combined with a dysmorphic syndrome, was found to be associated with *RYR1* mutations [134].

#### 3.4.3. Exertional Rhabdomyolysis

Recently, mutations in *RYR1* were found in one-third of unexplained rhabdomyolysis and/or exertional myalgia [107]. Patients usually presented between 3 and 45 years of age with rhabdomyolysis and/or exertional myalgia (*n* = 12), whereas isolated exertional myalgia was much less frequent. The most frequent triggers for rhabdomyolysis were exercise and heat [107]. Muscle strength was normal, and muscle biopsies revealed non-specific changes in most of the cases. *RYR1* mutations/variants detected in these cases have been previously reported in MHS or were localized to known MHS mutational hotspots, although only one out of the 24 reported patients had the MHS history [107].

#### 3.4.4. Other Ryr1-Related Phenotypes

Predominant axial myopathy had been reported in 12 *RYR1*-mutated patients, presenting between the third and eighth decade of life and suggesting *RYR1* as a possible cause of idiopathic camptocormia or bent spine syndrome [108,135]. Serum creatine kinase levels were normal or moderately elevated, and muscle imaging revealed the involvement of the lower paravertebral muscles and the posterior thigh. Muscle biopsy findings were described as discrete, with cores rarely reported. As for exertional rhabdomyolysis, *RYR1* sequencing revealed heterozygous missense variants previously associated with MHS trait or localizing to known MHS mutational hotspots.

Dominant or recessive RYR1 mutations have been detected in four patients with late-onset atypical PP, both with and without congenital myopathy [112,136]. Myalgia and cramps were prominent features in all the patients. In this regard, the direct interaction between the Cav1.1 channel and Ryr1 in the context of the excitation–contraction coupling makes the overlapping phenotypes when one of them is mutated, manifested by episodes of paralysis or congenital myopathy. Of note, marked impairment of the normal Cav1.1– Ryr1 interaction was observed in *RYR1*-related PP and recessive type myopathies [110,136].

### 3.5. RYR3-Related Myopathy with Nemaline Bodies

The RyR3 isoform expression and its active role in mammalian muscle contractility after post-natal age are unclear [137,138]. To date, there is only one report describing the mutation in the RyR3 gene in a 22-year-old woman affected by nemaline myopathy (NEM3) [139]. The patient showed dysmorphic facial features (high-arched palate, facial weakness, and micrognathia), proximal weakness of lower limbs starting at the age of five, and normal CPK but the myopathic pattern in electromyography. Skeletal muscle biopsy revealed predominant atrophic type 1 fibers, increased internal nuclei, and abundant nemaline bodies. She presented compound heterozygous missense mutations in *RYR3*, which are classified as variants of uncertain significance, as the role of RyR3 in muscle pathology is yet to be clarified [139].

### 3.6. TRPV4 Channel Related Myopathies

Among the transient receptor potential (TRP) cation channels, the vanilloid subtype TRPV4 is one of the most abundantly expressed in skeletal muscle [140]. In mouse muscles, it was found in a fraction of muscle fibers located at the sarcolemma and myonuclei. The TRPV4 protein is ubiquitously expressed and forms calcium-permeable channels opened by cell swelling, mechanical stimuli, and a variety of endogenous lipids. Physiologically, it may contribute to mechanosensitive ion channels, and its activation may attenuate fatigue by allowing Ca^2+^ influx [141,142]. Mutations in *TRPV4* can induce a number of peripheral neuropathies with implications for skeletal muscles, such as hereditary motor and sensory neuropathy type 2C mainly presenting in the pediatric age (HMSN2C), scapuloperoneal spinal muscular atrophy (SPSMA), and congenital distal SMA [143,144,145,146]. The genotype-phenotype relationship appears quite complicated. First, a number of asymptomatic carriers have been observed in several affected kindred, suggesting a reduced penetrance of some mutations. Second, *TRPV4* mutations can also cause skeletal dysplasias [147,148]. Surprisingly, a mutation previously found in a patient affected by autosomal dominant brachyolmia was detected in a patient suffering from HMSN2 [146,147]. Thus, it is now widely acknowledged that TRPV4 channelopathies can present with great phenotypic variability and overlapping features [149,150,151,152,153,154]. Interestingly, many of the mutations reported so far affect highly-conserved arginine residues located in the N-terminal ankyrin repeat domain (ARD), which is involved in oligomerization, intracellular trafficking, and interaction with other proteins [146,155]. Mutations located in other parts of the protein have been reported. The *TRPV4* mutations linked to skeletal dysplasia were shown to cause a gain of function of the channel [147,148]. Regarding neuropathy-causing mutations, various molecular defects have been proposed, including the gain of function, loss of function, and formation of intracellular aggregates with reduced plasma membrane expression. Very recently, in vivo experiments in Drosophila and primary mouse neurons showed that the expression of a *TRPV4* mutant causes neuronal dysfunction and axonal degeneration [156]. These effects were due to increased intracellular Ca^2+^ concentration, activation of a Ca^2+^/calmodulin-dependent protein kinase II, and impairment of axonal mitochondrial transport. Importantly, these effects were inhibited by TRPV4 blockers, which may suggest a promising therapeutic strategy. Noteworthy, little is known about the contribution of skeletal muscle TRPV4 channels to the diseases.

Besides peripheral neuropathies, a very recent study elegantly showed that skeletal muscle TRPV4 channels are involved in mechanically induced myotonia because the latter is inhibited by TRPV4 inhibitors or missing in muscles of TRPV4^−/−^ mice [157]. The authors proposed that mechanical stimulation of muscle fibers induces the opening of TRPV4 channels, leading to sarcolemma depolarization that is normally dampened by ClC-1 chloride currents. In myotonic muscles, the defective ClC-1 chloride channel cannot counteract the TRPV4-induced depolarization that, in turn, triggers hyperexcitability [157].

All these studies suggest that TRPV4 blockers may provide promising therapies. Such drugs are currently in development and have proven to be safe and well-tolerated in humans [158].

### 3.7. Congenital Amyotrophy Related to CACNA1H (T-Type Channel)

Very recently, a female baby presenting with severe amyotrophy at birth was reported [159]. Whole exome sequencing revealed the presence of two compound heterozygous mutations in the *CACNA1H* gene, which encodes a T-type voltage-gated calcium channel (Cav3.2). This channel is expressed in central and peripheral nervous systems, including motor neurons, neuroendocrine glands, smooth muscles, heart and kidney. Mutations in this gene have been clearly linked to primary hyperaldosteronism, while polymorphisms may increase susceptibility to childhood absence epilepsy and idiopathic generalized epilepsy. In these diseases, the pathomechanism is likely explained by a gain of function in the Cav3.2 channel. Conversely, functional study of mutations causing congenital amyotrophy suggested a loss of function. Since the channel may be involved in myoblast differentiation and fusion during embryogenesis, it is possible that loss of function impaired normal muscle development.

## 4. Potassium Channel-Related Myopathies

**Figure 3:**
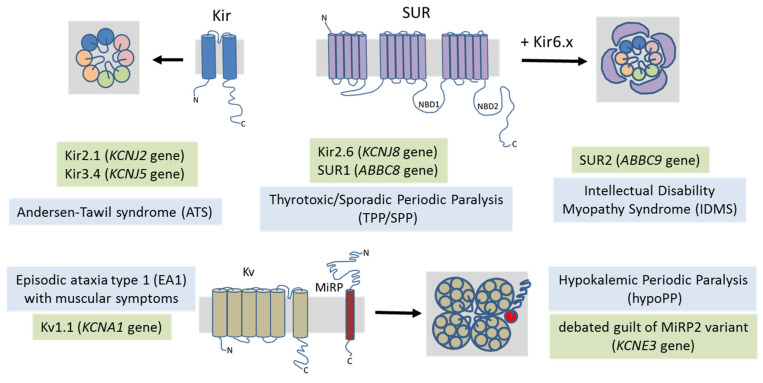
A schematic representation of the potassium channels involved in skeletal muscle myopathies. Potassium channel subunits are represented in lateral view embedded in the membrane, while multimeric assemblies are represented from the top view. The inward-rectifier K^+^ channels are heterotetrameric assemblies of Kir subunits. The ATP-sensitive K^+^ channels are made by the octameric association of four Kir6.x subunits with four sulfonylurea receptors (SUR) containing nucleotide-binding domains (NBD). The voltage-dependent K^+^ channel Kv1.1 is expressed in neurons, but a number of EA1 patients may present with predominant muscular phenotypes. The pathogenicity of the K^+^ channel auxiliary subunit MirP2 in hypoPP is still debated.

Potassium channels constitute the larger and most variegated family of ion channels, being involved in most cell functions. Thus, mutations in K channel genes have been linked to many diseases affecting many organs and, in some cases, to multisystem diseases (Figure 3).

### 4.1. Andersen–Tawil Syndrome

Andersen–Tawil syndrome (ATS) is a very rare autosomal dominant disorder (prevalence of 1 per 1,000,000) characterized by a triad of PP, cardiac arrhythmias, and typical facial and skeletal malformations [160,161]. Cardiac arrhythmias include ventricular arrhythmia, prolonged QT interval at the ECG, and prominent U waves. Malformations include ocular hypertelorism, low-set ears, small mandible, scoliosis, fifth digit clinodactyly, syndactyly, short stature, and a broad forehead [162]. However, a great variety of symptoms has been reported, making the diagnosis quite challenging. In addition, only one symptom of the triad may be present; hence, ATS should always be considered in patients presenting with only PP [163]. ATS usually presents within the first two decades of life. Regarding skeletal muscle, episodic weakness occurs with normo-, hyper-, or hypokalemia. Permanent muscle weakness was reported in a few cases [164,165,166,167]. Myokymia has been observed in one patient [168]. ATS is a potentially fatal disorder due to the occurrence of ventricular arrhythmia, and early diagnosis is required for proper management [41].

Mutations in *KCNJ2* encoding the inward-rectifying potassium channel Kir2.1 are responsible for about 60% of ATS cases (ATS type 1, also referred to as LQT7). This channel is expressed in skeletal muscle, heart, and bones, where it plays a role in stabilizing the resting membrane potential and contributes to cardiac action potential repolarizing phase. The ATS mutations induce a loss of function of Kir2.1 channels and consequent reduction of I_K1_ current in cardiomyocytes [169]. Many mutations are located in the binding site for phosphatidylinositol-4,5-biphosphate (PIP2), which is a known activator of Kir2.1 channels. Accordingly, several ATS mutations were shown to reduce PIP2 sensitivity [170,171,172,173]. However, other ATS mutations are distributed throughout the entire Kir2.1 protein, suggesting additional mechanisms. Indeed, some mutations were shown to impair intracellular trafficking [171,174]. Most of the ATS mutations exert a dominant negative effect on the wild-type channel subunit [169]. In addition, a mutation was shown to produce haploinsufficiency due to impaired synthesis or increased degradation [175].

Thus, the impairment of Kir2.1 channels logically accounts for the skeletal muscle and heart symptoms. In bones, experiments suggested that Kir2.1 is critical for osteoblastogenesis and bone morphogenetic protein signaling [176,177,178]. However, there is no clear correlation between the molecular defect and phenotypic variability, suggesting that other genes may influence the disease. One possible explanation resides in the capacity of Kir channels to form heteromultimeric channels [179,180]. Thus, the expression of the symptoms might depend on the expression balance between the various Kir subunits with respect to Kir2.1. Another possibility might be the occurrence of polymorphisms in other ion channel genes, including *KCNQ1*, *KCNH2* and *SCN5A*, which may influence the cardiac phenotype [181,182].

Noteworthy, gain-of-function mutations in *KCNJ2* have been linked to short QT syndrome [183,184].

Mutations of the *KCNJ5*-encoding Kir3.4 channel are found in about 15% of ATS cases (ATS type 2; LQT13) [162]. As for *KCNJ2*, *KCNJ5* mutations may have incomplete penetrance, and the expression of symptoms is variable [168,185,186]. This channel is responsible for the I_K(ACh)_ current in cardiomyocytes. A *KCNJ5* mutation was shown to reduce I_K(ACH)_ currents and to exert dominant negative effects on wild-type Kir3.4 and wild-type Kir2.1 channels [185,186].

The treatment of ATS may be challenging because of the need to address two distinct phenotypes—periodic paralysis and cardiac arrhythmias [161]. There is a risk that drugs beneficial to one organ may negatively affect the other. For cardiac arrhythmias, treatment includes the use of β-blockers such as propranolol. There are anecdotic observations of the positive effects of calcium or sodium channel blockers. In patients not responding to pharmacological treatment, the use of a pacemaker or an implantable defibrillator may be required. For PP, the treatment usually follows the guidelines for hypoPP or hyperPP, including the inhibitors of carbonic anhydrases such as ACZ and dichlorphenamide to reduce the frequency of attacks. However, muscle weakness exacerbation with ACZ has been reported in an ATS individual [187]. In the case of hypokalemic attacks, potassium supplementation may abort a paralytic attack while simultaneously shortening the QT interval. Potassium-sparing diuretics, such as spironolactone or triamterene, may be used in association with CAI or alone. In case of hyperkalemic attacks, the use of potassium-wasting diuretics, such as hydrochlorothiazide, should be considered very carefully due to the risk of a cardiac adverse effect of drug-induced hypokalemia.

### 4.2. Hypokalemic Periodic Paralysis

The *KCNE3* gene encodes MirP2, an auxiliary subunit of K channels. The missense p.R83H mutation in *KCNE3* was found in two pedigrees affected by periodic paralysis [188]. The same mutation was found in one of 14 patients affected by thyrotoxic periodic paralysis (TPP) [189]. However, the direct link between p.R83H and periodic paralysis was challenged by genetic studies performed on larger groups, showing a frequency similar in controls and patients as well as lacking segregation of the mutation with PP [190,191,192,193]. Thus, more studies are needed to verify whether *KCN3E* mutations cause hypoPP, constitute susceptibility polymorphisms, or are simply not related to hypoPP. Intriguingly, we found a reduced *KCNE3* mRNA level in muscle biopsies of two patients affected by recessive myotonia congenita (Becker’s disease), but an increase in one patient with dominant myotonia congenita (Thomsen’s disease), compared to control individuals [194,195].

### 4.3. Thyrotoxic Periodic Paralysis

TPP linked to hyperthyroidism is the most common cause of hypokalemic flaccid muscle weakness in adult Asian and Hispanic males [196]. Of note, TPP is often indistinguishable from hypoPP; thus, an assessment of thyroid function should be always performed in patients with hypoPP. Besides, sporadic periodic paralysis (SPP) is characterized by normal thyroid function and lack of *CACNA1S*, *SCN4A*, or *KCNJ2* mutation. Nevertheless, TTP and SPP are closely related since they appear to share a number of susceptibility genes [197,198,199,200]. Mutations in the *KCNJ18* gene have been reported in a few cases with TPP [201,202,203,204]. The *KCNJ18* gene encodes the skeletal muscle inward-rectifier K channel Kir2.6, the expression of which is enhanced by thyroid hormone (T_3_) through a promoter response element. Several *KCNJ18* variants were shown to reduce Kir2.6 currents by affecting single-channel conductance, open probability, or cell surface expression. Some of these effects were T_3_-dependent, including altered modulation by PIP2 and PKC, which are activated during thyrotoxicosis. In addition, some Kir2.6 mutants exert a dominant negative effect not only on wild-type Kir2.6 but also on Kir2.1 channels, where the mutation is responsible for ATS1 [202]. It was proposed that wild-type Kir2.6 channels are activated by T_3_ in healthy muscle to maintain the resting membrane potential, thereby balancing the T_3_-induced weakness. Consequently, the loss-of-function mutations in *KCNJ18* may increase the susceptibility of muscle to thyrotoxicosis-induced weakness. Interestingly, other susceptibility genes were identified in both TTP and SPP on chromosome 17q close to the *KCNJ2* gene, suggesting a possible influence on Kir2.1 channel expression [197,205,206,207]. In addition, a variant of the ***ABCC8*** gene encoding SUR1, the sulfonylurea receptor type 1 associated with ATP-sensitive inward-rectifier K channels (K_ATP_), was proposed as another susceptibility gene for TPP [208]. Thus, it appears that the pathomechanism of TPP and SPP may be similar to that found in ATS. Nonetheless, many questions are still open, and more studies are needed to confirm the genotype–phenotype relationship [204,209,210].

### 4.4. Intellectual Disability Myopathy Syndrome

Recently, a new disease named intellectual disability myopathy syndrome (IDMS) was described in two pedigrees from Norway [211]. The disease is multisystemic, inducing intellectual disability, partial hearing loss, developmental delay, anxiety, sleep disorder, hyperreflexia, cerebral MRI anomalies, skeletal dysmorphism, cardiac systolic dysfunction, and myopathy. The muscle symptoms include generalized hypotonia in childhood and weakness and fatigability in adults. A muscle biopsy performed in one patient showed variations in fiber diameter and the presence of mitochondria aggregates. A splice-site mutation was identified in the *ABCC9* gene that codes for SUR2, the sulfonylurea receptor type 2. From genetic databases, it appeared that, in the heterozygous state, the variant frequency was higher in the Finnish population compared to other Europeans and was absent outside Europe. All the patients were homozygous for the variant, in accord with the recessive inheritance. The SUR2 variant induced a complete loss of functional K_ATP_ channels in cells co-expressing Kir6.2. In agreement with the inheritance trait, the mutant does not exert any dominant negative effect on WT SUR2. In mice, the insertion of a premature stop codon in *ABCC9*, which induced a loss of function of SUR2, jeopardized the physical performance due to increased fatigability in patients. Differences in muscle symptoms associated with SUR1 (TPP) and SUR2 variants are quite surprising since both subunits bind to Kir6.2 to form K_ATP_ channels. However, the relative abundance of SUR1 and SUR2 is probably muscle-type-specific, as shown in mice [212], and their contribution to mitochondrial KATP channels is not established yet [213]. Another important difference between ATS and IDMS regards the inheritance mode of SUR1 and SUR2 mutations. Thus, as for most of the muscle ion channelopathies, recessive mutations are more threatening than dominant ones.

### 4.5. Episodic Ataxia/Myokymia

The episodic ataxias are a heterogeneous group of autosomal dominant diseases characterized by recurring attacks of cerebellar ataxia. Episodic ataxia type 1 (EA1) is caused by mutations in the *KCNA1* gene encoding the voltage-dependent potassium Kv1.1 channel [214,215]. EA1 shows a broad phenotypic spectrum, including cerebellar ataxia, muscle spasticity, dyskinesia, seizures, epileptic encephalopathy, hyperthermia, and hypomagnesemia [216,217]. Muscle symptoms include constant myokymia and acute episodes of uncontrolled muscle contractions of the face and limbs, stiffness, cramps, and weakness, as well as malignant hyperthermia susceptibility in one kindred. In a few patients, muscle symptoms are largely preponderant. Functional studies of *KCNA1* mutations in heterologous systems of expression indicate a loss of function of the Kv1.1 channel through altered gating, expression, or modulation. Studies of a knock-in mouse EA1 model provided mechanistic insights for the disease [218]. Loss of function in Kv1.1 increases the excitability of basket cells to release GABA that, in turn, inhibits Purkinje cells in the cerebellum, thereby inducing ataxia [218,219]. Mutations linked to ataxia are spanned over the entire protein. However, the loss of function of juxtaparanodal Kv1.1 channels increases the motor nerve excitability, which promotes myokymia [220]. Interestingly, mutations causing preeminent muscle symptoms with little or no ataxia are clustered in the transmembrane S2 and S3 helices and the S2–S3 intracellular linker [195,221,222,223]. Since K_V_1.1 channels are expressed in many central neurons, loss-of-function mutations may induce excessive brain excitability leading to epilepsy. Mutations affecting the channel pore region appear especially inclined to induce seizures. Those mutations in the conserved Pro-Val-Pro motif in the sixth transmembrane segment are linked to epileptic encephalopathy, i.e., recurrent seizures and intellectual disability [224,225]. The selective deletion of *KCNA1* in neurons causes epilepsy, premature death, and cardiorespiratory dysregulation in mice [226]. The variability of symptoms associated with *KCNA1* mutations suggests that mutations may manifest their effects in a cell type-dependent manner. Such effects might depend on the specific biophysical defect or on specific interactions with other proteins, such as other Kv subunits. In addition, environmental factors and gene modifiers may account for the different symptoms observed in patients carrying the same mutation [195,217]. Such a great variability in symptoms makes *KCNA1*-related diseases difficult to diagnose. For instance, next-generation sequencing recently revealed an already known *KCNA1* mutation (p.N255D) in a patient diagnosed with myotonia, presenting with hand and facial stiffness without ataxia [227].

Pharmacological therapy of *KCNA1*-related diseases is symptomatic only [216,228,229]. The carbonic anhydrase inhibitor ACZ provided some relief in a number of patients. However, limiting side effects can develop in the long term (kidney stones, excessive sweating, paresthesia, muscle stiffness and fatigability, gastrointestinal disorders, concentration and memory difficulties). In addition, non-responders have been frequently observed. The mechanism of action of ACZ in EA1 and other ion channelopathies is still unclear. Whether Kv1.1 channels are sensitive to the change in pH value is unknown. Antiepileptic drugs, including sodium channel blockers (carbamazepine, oxcarbazepine, lamotrigine, phenytoin, valproic acid), phenobarbital, benzodiazepine (clonazepam, clozapam), vigabatrin, and gabapentin, have been used with some benefit to relieve ataxia and seizures. In some patients, these drugs may be ineffective or may worsen symptoms. However, there is no drug-class effect, as a patient refractory to one or more antiepileptic drugs may have benefited from another. No information is available regarding the effects of these drugs on wild-type or mutated Kv1.1 channels. In addition, information is lacking regarding the effects of treatment on muscle symptoms.

## 5. Chloride Channel-Related Muscle Disorders

The chloride channel family includes a variety of proteins involved in many different cellular functions, which include maintenance of ion homeostasis, trans-epithelial fluid transport, modulation of intracellular compartment pH value, cell volume regulation, and the regulation of cell excitability [230]. In skeletal muscle fibers, the high level of sarcolemma chloride conductance (g_Cl_) is crucial for the stabilization of the resting membrane potential and the repolarization of action potentials [231].

The gCl in skeletal muscle is mediated by the voltage-gated ClC-1 chloride channels. It was the first mammalian voltage-gated chloride-channel identified belonging to the family of CLC proteins [232].

With a mass of ~120 kDa, ClC-1 is the largest protein of this family, composed of two identical subunits, each forming a gated pore. Each subunit consists of 18 α-helices, 17 of which are embedded in the plasma membrane, and 2 tandem cystathionine-β-synthase (CBS) domains located in the intracellular C-terminal region [233,234].

Loss-of-function mutations in the *CLCN1* gene abolish or reduce ClC-1 plasma membrane currents, leading to myotonia congenita (MC), the most frequent hereditary skeletal muscle channelopathy in humans [2,235,236]. MC can be inherited either as an autosomal dominant (Thomsen’s disease) or an autosomal recessive (Becker’s disease) trait; Becker’s disease is associated with a more severe phenotype. MC patients present with muscle stiffness and delayed muscle relaxation after a voluntary movement, a clinical sign called myotonia. Myotonia in MC is worse at rest and instead improves with exercise, the so-called warm-up phenomenon [42]. Other symptoms include muscle hypertrophy, more frequent in Becker’s disease and conferring a “hercules” habitus to the patient, and cold sensitivity of myotonia, although less frequent than in patients carrying *SCN4A* mutations. Myotonia in MC is more frequently observed in the limbs, while cranial myotonia is much more suggestive of SCM or PMC [4,11,237]. Painful myotonia is more frequent in SCM than in MC [11,237]. Furthermore, patients with Becker’s disease may experience transient weakness at the onset of voluntary contraction that may rarely lead to falls [238].

More than 250 *CLCN1* mutations have been found to be associated with MC. They scattered over the entire sequence of the channel protein, but a few regions with high-frequency mutations have been described in exons 4, 5, and 8 [2,239,240,241]. With the exception of a few nonsense mutations leading to truncated ClC-1 proteins, all dominant mutations are missense mutations. Recessive mutations include deletions, insertions, splice-site, missense, or nonsense errors. In addition, a combination of whole exon deletions or duplications and point mutations in *CLCN1* accounted for 6% of patients with MC [242].

**Figure 4:**
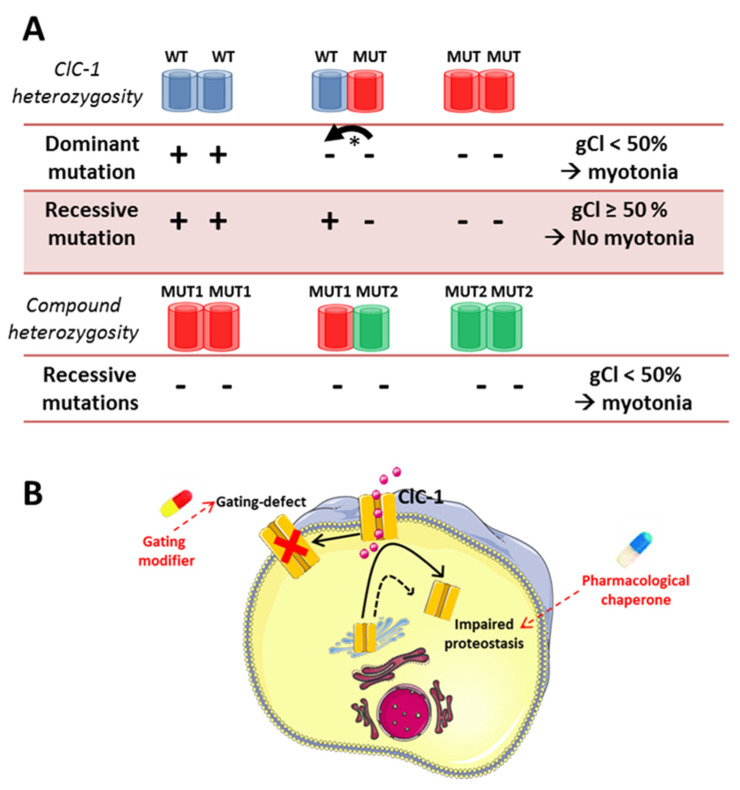
Pathomechanisms of CLCN1-related myotonia congenita (MC). (**A**) Presumed mechanism of inheritance in MC. The ClC-1 channel is a dimeric protein with a double barrel-like structure: two subunits assemble to form two parallel ion-conducting pores. In the case of heterozygosity, wild-type (WT) and mutated (MUT) ClC-1 subunits can assemble as homodimers, WT–WT and MUT–MUT, and heterodimers (WT–MUT). In autosomal dominant Thomsen’s disease, the mutated subunit is expected to exert a dominant-negative effect (*) on the associated wild-type subunit in the WT–MUT channel. This results in the reduction of sarcolemma chloride conductance (gCl) by more than 50%, leading to muscle fiber hyperexcitability and muscle stiffness. In autosomal recessive Becker’s disease, the recessive mutation is expected to have no effect on the associated WT subunit. The co-expression of the recessive mutation with the wild-type ClC-1 results at maximum in a 50% reduction of the sarcolemmal chloride conductance, which is not enough to cause myotonia. The occurrence of recessive mutations in homozygosity or in compound heterozygosity is required to reduce the sarcolemmal chloride conductance by more than 50% and to induce myotonia. (+: functional ClC-1 subunit, -: impaired ClC-1 subunit). (**B**). The main molecular defects of ClC-1 mutants associated with MC consist of either a defect in ClC-1 proteostasis (protein synthesis, cell trafficking, or proteolysis) or a defect in ClC-1 channel gating. Knowledge of molecular pathomechanism may guide the development of efficient mutant-targeted pharmacological treatments to restore ClC-1 function.

In vitro functional studies of ClC-1 mutations have clarified the molecular mechanisms by which the dominant and recessive mutations alter ion channel function and expression (Figure 4). In Thomsen’s disease, the mutant subunit is expected to exert a dominant-negative effect on the associated wild-type (WT) subunit in the dimeric channel [243]. Thus, in the heterozygous carrier, the gCl is reduced by more than 50%, leading to overexcitability. This is the case of the p.T335N mutation, identified for the first time in an Italian family. This mutation induced a reduction of chloride current and exerted a dominant negative effect on the WT subunit, consistent with mild Thomsen’s phenotype [244]. In contrast, ClC-1 mutated subunits associated with recessive myotonia do not exert a significant dominant-negative effect on the associated subunit. In the heterozygous carrier, WT subunits sustain at least 50% of the gCl, and muscle function is preserved. In the compound heterozygous carrier, all the subunits are mutated, and the gCl is reduced 50% more, leading to myotonia [243].

The majority of MC mutations are predicted to reduce chloride currents by impaired ClC-1 proteostasis (impaired synthesis, defective cell trafficking, increased degradation) or by impairing ClC-1 function through various gating defects. The latter one includes a positive shift of the voltage-dependent activation of fast and slow channel gating, reduction of single-channel conductance, altered ion selectivity, or an inverted voltage dependence (hyperpolarization-activated) [241,244,245,246,247,248,249]. A number of ClC-1 mutations found in myotonic patients show no evidence of any functional defect when studied in heterologous expression systems. Notable examples include several missense mutations, such as p.T82A, p.F167L, p.R453W, p.L628P, and p.L861P [28,194,246,247]. This suggests that the defect of these variants may arise only in the skeletal muscle cells due to altered interactions with muscle proteins.

Currently, MC patients receive symptomatic treatment, which aims at dampening skeletal muscle hyperexcitability through inhibition of voltage-gated sodium channels. Thus, mexiletine was employed as the first-line therapy in MC, similarly to sodium channel myotonia and paramyotonia congenita [22]. Very recently, persistent sodium currents have been proposed to be involved in the mechanism underlying the transient weakness in recessive MC [250]. This may explain why mexiletine is greatly efficient against this debilitating symptom [238]. Similar to PMC and SCM, lamotrigine can be considered as a second choice in MC [26,27].

Besides sodium channel blockers, ACZ was reported to be efficient in small cohorts of MC patients [251,252] and could be considered a second choice. The mechanism of ACZ is not well defined, but it might stimulate the ClC-1 channel activity through intracellular acidification [39,253]. However, there is no known direct activator of ClC-1 available. The classification of ClC-1 mutations according to their functional defect would be fundamental to help the discovery of new drugs that target mutant channels’ defects with the hope of ensuring a personalized treatment for MC patients. This includes the drug able to correct mutant channel gating (gating-modifiers) or to improve the expression of traffic-defective mutants (pharmacological chaperones) [231].

## 6. Nicotinic Receptor-Channels

Nicotinic acetylcholine receptor (AChR) is localized on the crests of the folded postsynaptic muscle membrane. The binding of acetylcholine with its receptor opens the central ion channel pore of the AChR, resulting in depolarization of the muscle membrane. The AChR is a pentamer comprising four different subunits (α, β, δ, ε in a ratio of 2:1:1:1), with each subunit encoded by a separate gene and forming a transmembrane ligand-gated ion channel. Congenital myasthenic syndrome (CMS) is a rare genetic disorder due to mutations in genes encoding proteins involved in the neuromuscular junction (NMJ) structure and function, causing skeletal muscle weakness and fatigability [254]. CMS may be classified based on the localization in the NMJ or the function of the mutated protein, with AChR defects accounting for around half of the CMS cases [255]. CMS related to AChR defects includes AChR deficiency and, much less frequently, AChR kinetic defects, which can be classified into slow and fast-channel syndromes. All the AChR subunits may be involved in CMS, especially the epsilon subunit gene (*CHRNE*) is mutated in about 20%–50% of the cases [255,256,257]. CMS related to AChR defects are recessively inherited, except for slow-channel syndrome (SCS), which is an autosomal dominant disease [254].

The clinical presentation of CMS due to AChR defects is highly variable, ranging from mild symptoms to severe manifestations, sometimes with life-threatening respiratory episodes, especially in the first decade of life. These CMS subtypes usually present at birth or in infancy, often with hypotonia in association with ocular, facial, bulbar or respiratory symptoms and followed by delayed motor milestones. Alternatively, they may present later with walking difficulty and frequent falls. Prenatal onset with arthrogryposis multiplex congenital (AMC) and fetal akinesia (FA) was originally reported on patients mutated in *CHRNG*, coding for the gamma subunit of the fetal form of AChR [258]. No myasthenic symptoms were observed at birth or later because the adult AChR was normally expressed. AMC and FA have been reported in association with mutations in other CMS genes, including *CHRNA1* and *CHRND*, characterized by CMS symptoms and absence of adult AChR in the disease animal model [259,260]. CMS is usually characterized by static or slowly progressive course over the years, although acute exacerbations may be triggered by infectious episodes, surgery, or stress, sometimes leading to acute respiratory failure, in particular in the first years of life [254]. The central nervous system and heart are not involved. The CMS diagnosis is based on clinical and electromyography findings, followed by the identification of the molecular defect, which may be guided by specific clinical, neurophysiological or histological clues [254].

Only symptomatic pharmacological therapies are available for CMS treatment. Of note, drugs with positive effects on specific forms of CMS may worsen another one, as pyridostigmine in SCS. Hence, genetic diagnosis is highly recommended to optimize pharmacologic treatment. Drugs useful to treat CMS due to AChR defects are mainly cholinergic agonists (PYR and 3,4 DAP). Adrenergic agonists (salbutamol/albuterol and ephedrine) may be used in patients not responsive to cholinergic agonists. Fluoxetine and quinidine are long-lived open-channel blockers of the AChR ion channel, and they are currently used only in SCS [254]. Salbutamol may be used even in SCS [261].

## 7. Concluding Remarks

The SMICs represent an expanding field; in this regard, new genes and new phenotypes have been recently discovered after the introduction of next-generation sequencing [227,262,263,264,265]. Clinical variability warrants better comprehension of genotype–phenotype correlations and of other mechanisms influencing clinical expression [266]. For instance, the coexistence of mutations in two different genes and non-genetic factors (epigenetic, environmental, and hormonal) may help the understanding of the clinical variability [24,267,268,269].

Up to date, most treatments are only symptomatic. However, knowledge of the pathomechanisms and detailed phenotyping may offer the possibility to develop more targeted therapies with increased efficacy and safety. Early molecular diagnosis is critical because the choice of therapeutic option may help save lives or slow down the disease progression. Ion channels are expressed on the membrane and may be easily druggable with small molecular weight molecules or monoclonal antibodies. Small compounds able to correct the specific defect induced by mutations are warranted, as it occurred for the pharmacological treatment of CFTR mutations in cystic fibrosis [270]. To this purpose, an interdisciplinary approach including both clinical, molecular, neurophysiological and pharmacological data is the only promising strategy to find efficient new treatments for SMICs.

## Figures and Tables

**Table 1:** Ion channels gene mutations and related clinical muscular phenotypes.

Protein	Gene	Inheritance	Clinical Phenotype	Phenotype MIM	Age at Onset	Muscle Biopsy	Specific Features	Treatment
SODIUM CHANNEL SUBUNITS								
Nav1.4	SCN4A	AD	Sodium channel myotonia (SCM; includes potassium-aggravated myotonia, myotonia fluctuans, myotonia permanens, ACZ-responsive myotonia, and SNEL)	608,390	Highly variable (neonatal–early childhood–adulthood)	non-specific myopathic pattern	Predominance in cranial muscles, precipitated by cold, presence of warm-up, muscle weakness absent or mild with late-onset	Mexiletine, lamotrigine, carbamazepine, ACZ, flecainide, and propafenone
		AD	Paramyotonia congenita (PMC)	168,300	First decade	non-specific myopathic pattern	Myotonia and episodic muscle weakness precipitated by cold, paradoxical myotonia, predominant in cranial muscles, possible fixed muscle weakness in late disease stages.	As for SCM
		AD	Hyperkalemic periodic paralysis (hyperPP)	170,500	First decade	Vacuolar and tubular aggregate myopathy	Episodic flaccid muscle weakness lasting up to 2 h accompanied by hyperkalemia > 4.5 mEq/L. Associated with myotonia.	ACZ, diclorophenamide
		AD	Hypokalemic periodic paralysis type 2 (hypoPP2)	613,345	Childhood–third decade	Vacuolar and tubular aggregate myopathy	Episodic flaccid muscle weakness lasting up to 24 h accompanied by hypokalemia < 3.5 mEq/L.	ACZ, K^+^ sparing diuretics (spironolactone, triamterene)
		AR	Congenital myasthenic syndrome type 16 (CMS16)	614,198	Neonatal or early infancy	non-specific myopathic pattern	Decremental response of the CMAP on RNS. Predominant involvement of bulbar and respiratory muscles.	Pyridostigmine and ACZ may be beneficial
		AR	Congenital myopathy	n.a.	Neonatal or early infancy	No evident nemaline rods or structural abnormalities	Predominant axial and pelvic muscle weakness, delayed motor milestones, improvement in strength over time	ACZ
CALCIUM CHANNEL SUBUNITS								
Cav1.1	CACNA1S	AD	Hypokalemic periodic paralysis type 1 (hypoPP1)	170,400	Childhood–second decade	Vacuolar myopathy (non-rimmed)	Episodic flaccid muscle weakness lasting up to 24 h accompanied by hypokalemia < 3.5 mEq/L. Fixed myopathy often developing in late disease stages.	ACZ, diclorphenamide
		AD	Malignant hyperthermia susceptibility type 5 (MHS5)	601,887	When exposed to volatile anesthetics or succinylcholine	No structural abnormalities	MHS	Dantrolene (antidote)
		AR	Congenital myopathy	n.a.	Neonatal or early infancy	Centronuclear or core myopathy	Hypotonia, delayed motor milestones, facial involvement (ophthalmoplegia), progressive muscle weakness (mainly axial).	ACZ
Stac3	STAC3	AR	Bailey-Bloch congenital myopathy/Native American Myopathy (NAM)	255,995	Neonatal or early infancy	non-specific myopathic pattern	Dysmorphic facial features and facial weakness (ptosis) susceptibility to MHS; multiple joint contractures	n.a.
Orai 1	ORAI1	AD	Tubular aggregate myopathy (TAM2)	615,883	Childhood	tubular aggregates in type II fibers, predominance of type I fibers	Slowly progressive	n.a
		AR	CRAC channelopathies	612,782	<1 year	(1 patient) myopathic pattern; no evident nemaline rods or structural abnormalities.	Congenital non-progressive myopathy; immunodeficiency as the main feature.	n.a.
Stim 1	STIM1	AD	Stormorken syndrome (STRMK), York platelet syndrome	185,070	Childhood–early adult	tubular aggregates in type II fibers, predominance of type I fibers	Thrombocytopenia, anemia, asplenia, congenital miosis, and ichthyosis, asymptomatic to slowly progressive proximal muscle weakness	n.a.
		AD	Non-syndromic tubular aggregate myopathy (TAM1)	160,565	Childhood	tubular aggregates mainly in type II fibers; predominance type I fibers	Slowly progressive, elevated CPK	n.a.
		AR	CRAC channelopathies	612,783	<1 year	n.a.	Congenital non-progressive myopathy; immunodeficiency as the main feature.	n.a.
Ryr1	RYR1	AD	Malignant hyperthermia susceptibility type 1 (MHS1)	145,600	When exposed to volatile anesthetics or depolarizing muscle relaxants	Central core, multiminicore myopathy	MHS	Dantrolene (antidote)
		AD/AR	Central core disease	117,000	First decade, rarely in adulthood	Central cores in type 1 fibers, predominance of type 1 fibers	Floppy infant; non-progressive/slowly progressive myopathy; joint contractures	n.a
		AR	Multiminicore myopathy with external ophthalmoplegia	255,320	Neonatal or early infancy	Dystrophic signs and minicores	Hypotonia, delayed motor milestones, dysmorphic facial features and facial weakness, and progressive muscle weakness	n.a
Ryr3	RYR3	AR	Nemaline myopathy (NEM3)	161,800	1 case, infantile	Perinuclear and subsarcolemmal nemaline bodies, wide variation in fiber size with type 1 fiber predominance and atrophy, increased internal nuclei	Dysmorphic face; normal CPK; myopathic EMG. Slowly progressive proximal limb weakness	n.a.
TRPV4	TRPV4	AD (reduced penetrance)	scapuloperoneal spinal muscular atrophy (SPSMA)	181,405	Neonatal or early infancy	Grouped type 1 and 2 fiber atrophy	Non-progressive or slowly progressive scapular and peroneal muscle weakness and atrophy, and peripheral motor neuropathy	n.a.
		AD (reduced penetrance)	congenital distal spinal muscular atrophy (CDSMA)	600,175	Neonatal or early infancy	neurogenic muscle damage	Variable from lower-limb muscle weakness to severe neurogenic weakness and arthrogryposis	n.a.
		AD	Hereditary motor ans sensory neuropathy (HMSN2C)	606,071	Variable (infancy, childhood, adulthood)	Neurogenic muscle damage and atrophy	Axonal polyneuropathy, diaphragmatic and vocal cord paresis, and distal muscle weakness	n.a.
Cav3.2	CACNA1H	AR	Congenital amyotrophy		1 case, neonatal	n.a.	Severe amyotrophy at birth	n.a.
POTASSIUM CHANNEL SUBUNITS								
Kir2.1	KCNJ2	AD	Andersen–Tawil syndrome type 1 (ATS1, LQT7)	170,390	Variable onset of periodic paralysis (early childhood-adulthood)	non-specific myopathic pattern (few cases)	Potassium-sensitive periodic paralysis, cardiac arrhythmia, and facial and skeletal malformations. Prolonged QT (plus other arrhythmias). Episodic flaccid paralysis lasting up to 24h and more, usually accompanied by hypokalemia.	ACZ, dichlorphenamide, ß-blockers, If hypokaliemic, K+ supplementation or K+ sparing diuretics. If hyperkaliemic, be careful with K+ wasting diuretics for cardiac risk.
Kir3.4	KCNJ5	AD	Andersen–Tawil syndrome type 2 (ATS2, LQT13)	613,785	Variable onset of periodic paralysis as for ATS1	n.a.	Same triad of symptoms of ATS1	As for ATS1
Kir2.6	KCNJ18	AD	Susceptibility to thyrotoxic periodic paralysis type 2 (TTPP2)	613,239	Early adulthood	Variable, vacuolation, mitochondrial changes, glycogen granules accumulation.	Episodic flaccid paralysis during thyrotoxicosis and hypokalemia. More frequent in Asian males.	Treatment of hyperthyroidism
Sur2	ABCC9	AR	Intellectual disability myopathy syndrome (IDMS)	n.a.	Variable onset of muscle symptoms (childhood-adulthood)	(1 patient) non-specific changes (mitochondrial aggregation, fiber caliber variation)	Hypotonia, muscle weakness and fatigability, dysmorphic features, intellectual disability and developmental delay, and cardiac systolic dysfunction	n.a.
Kv1.1	KCNA1	AD	Episodic ataxia/myokymia syndrome (EA1)	160,120	Childhood	non-specific, denervation findings	Muscle symptoms (preponderant in a few patients): constant myokymia and acute episodes of muscle contractions (face and limbs), stiffness, cramps, weakness, and episodic cerebellar ataxia, seizures, and hypomagnesemia.	ACZ antiepileptic drugs
CHLORIDE CHANNEL SUBUNITS								
ClC-1	CLCN1	AD	Myotonia congenita, Thomsen’s disease	160,800	First decade	No structural abnormalities	Myotonia with the warm-up phenomenon, cold sensitivity, predominant in limb muscles.	Mexiletine lamotrigine, carbamazepine, ACZ
		AR	Recessive generalized myotonia, Becker’s disease	255,700	First decade	No structural abnormalities	More severe than Thomsen’s disease; transient weakness and “Herculean” appearance	As for Thomsen’s disease
NICOTINIC RECEPTOR								
AChR	CHRNE	AR	Congenital myasthenic syndrome (CMS4C) associated with AChR deficiency	608,931	Variable, infancy–adulthood	non-specific	Variable, from arthrogryposis multiplex congenital and fetal akinesia, ocular, bulbar and respiratory symptoms, delayed motor milestones, to mild adult muscle weakness. Slowly progressive with possible exacerbations. Decremental CMAP in response to RNS-EMG.	Pyridostigmine, 3,4 diaminopyridine, salbutamol/albuterol, and ephedrine
		AD/AR (rare)	Slow-channel CMS (CMS4A)	605,809	Infancy–young adult	non-specific	Neonatal hypotonia; ocular, bulbar, respiratory muscle involvement, with predominant weakness of cervical, wrist, finger, and finger extensor muscles. Double CMAP on single nerve stimuli ENG.	Quinidine, fluoxetine, Worsening with pyridostigmine
		AR	Fast-channel CMS (CMS4B)	616,324	Infancy	non-specific	Neonatal hypotonia, recurrent respiratory crises. Ocular, neck, and limb progressive muscle weakness and fatigability. Decremental CMAP in response to RNS-EMG.	Pyridostigmine, 3,4 diaminopyridine
	CHRNA1	AD	Slow-channel CMS (CMS1A)	601,462	Infancy–young adult	non-specific	Ocular, bulbar, and respiratory muscle involvement, with predominant weakness of cervical, wrist, finger, and finger extensor muscles. Double CMAP on single nerve stimuli ENG.	Quinidine, fluoxetine, Worsening with pyridostigmine
		AD (rare)/AR	Fast-channel CMS (CMS1B)	608,930	Infancy	non-specific	Neonatal hypotonia; ocular, bulbar (dysarthria), neck, recurrent respiratory crises, and limb progressive muscle weakness and fatigability. Decremental CMAP in response to RNS-EMG.	Pyridostigmine, 3,4 diaminopyridine
	CHRNB1	AR	CMS2C associated with AChR deficiency	616,314	Birth	non-specific	Neonatal hypotonia; respiratory and limb progressive muscle weakness. Decremental CMAP in response to RNS-EMG	Pyridostigmine
		AD	Slow-channel CMS2A	616,313	Infancy–young adult	non-specific	Ocular, bulbar, respiratory muscle involvement, with predominant weakness of cervical, wrist, finger, and finger extensor muscles. Double CMAP on single nerve stimuli ENG.	Quinidine, fluoxetine, Worsening with pyridostigmine
	CHRND	AD	Slow-channel CMS3A	616,321	Infancy—young adult	non-specific	Ocular, bulbar, respiratory muscle involvement, with predominant weakness of cervical, wrist, finger, and finger extensor muscles. Double CMAP on single nerve stimuli ENG	Quinidine, fluoxetine, Worsening with pyridostigmine
		AR	CMS3C associated with AChR deficiency	616,323	Birth	non-specific	Neonatal hypotonia; ocular, episodic respiratory insufficiency, bulbar (swallowing), and proximal limb muscle weakness. Decremental CMAP in response to RNS-EMG.	Pyridostigmine
		AR	Fast-channel CMS3B	616,322	Infancy	non-specific	Neonatal hypotonia; ocular, neck, respiratory, muscle weakness and fatigability. Decremental CMAP in response to RNS-EMG.	Pyridostigmine, 3,4-diaminopyridine

References are given in the text. Abbreviations: ACZ: Acetazolamide; CMAP: Compound Muscle Action Potential; CPK: creatine phosphate kinase; n.a.: not available; RNS-EMG: Repetitive nerve stimulation electromyography.

## Data Availability

Not applicable.
